# Effectiveness of a vitamin D regimen in deficient multiple myeloma patients and its effect on peripheral neuropathy

**DOI:** 10.1007/s00520-023-07574-0

**Published:** 2023-01-26

**Authors:** Berdien E. Oortgiesen, Marloes Dekens, Ruud Stapel, Abdulrazzaq Alheraky, Pauline de Keizer Dannenberg, Claire Siemes, Frank G. A. Jansman, Robby E. Kibbelaar, Nic J. G. M. Veeger, Mels Hoogendoorn, Eric N. van Roon

**Affiliations:** 1 Department of Clinical Pharmacy and Pharmacology, Medical Centre Leeuwarden, P.O. Box 888, 8901 BR Leeuwarden, the Netherlands; 2 Department of Hematology, Deventer Hospital, Deventer, the Netherlands; 3 Unit of Pharmacotherapy, Epidemiology and Economics, Department of Pharmacy, University of Groningen, Groningen, the Netherlands; 4 Department of Clinical Pharmacy and Pharmacology, Deventer Hospital, Deventer, the Netherlands; 5 Department of Pathology, Pathology Friesland, Leeuwarden, the Netherlands; 6 Department of Epidemiology, MCL Academy, Leeuwarden, the Netherlands; 7 Department of Epidemiology, University of Groningen, University Medical Centre Groningen, Groningen, the Netherlands; 8 Department of Hematology, Medical Centre Leeuwarden, Leeuwarden, the Netherlands

**Keywords:** Multiple myeloma, Vitamin D deficiency, 25-Hydroxyvitamin D, Dose–response relationship, drug, Peripheral nervous system diseases

## Abstract

**Purpose:**

Peripheral neuropathy (PN) is common in multiple myeloma (MM) patients. More insight has been gained concerning the role of vitamin D in preventing PN. However, studies evaluating the effects of vitamin D_3_ supplementation on PN are lacking. The aims of this study are to (1) evaluate the effectiveness of a vitamin D_3_ regimen on achieving adequate vitamin D levels in deficient MM patients and to (2) exploratively evaluate the effect of vitamin D_3_ supplementation on PN.

**Methods:**

Thirty-nine MM patients with inadequate (< 75 nmol/L [= 30 ng/mL]) 25-hydroxyvitamin D (25(OH)D) levels were included in this multicenter, prospective, single-arm study, of whom 35 patients completed the study. They received oral vitamin D_3_ for 6 months according to a dose escalation regimen that consisted of one or two loading doses of 200,000 international units (IU), and maintenance doses of 800, 1600, or 3200 IU/day depending on the 25(OH)D level. A validated questionnaire was used to measure PN.

**Results:**

Median 25(OH)D increased from 38 (IQR 32–52) nmol/L at baseline to 77 (IQR 72–87) nmol/L after 6 months (*P* < 0.001). Adequate 25(OH)D levels were achieved by 66% of the subjects, and 34% were within the range of 50–75 nmol/L. Furthermore, in 37% of the participants, PN severity decreased (*P* = 0.007).

**Conclusion:**

The use of substantially higher vitamin D_3_ doses than recommended in current guidelines resulted in a significant increase in vitamin D levels in MM patients. Furthermore, evaluation of PN showed a significant decrease in PN grading. However, this exploratory evaluation needs further confirmatory research.

**Supplementary Information:**

The online version contains supplementary material available at 10.1007/s00520-023-07574-0.

## Introduction

Multiple myeloma (MM) is a malignant proliferation of plasma cells in the bone marrow that occurs among older adults, and accounts for approximately 10% of all hematological malignancies [1]. Bone disease due to displacement of the bone marrow environment by plasma cells is one of the major problems in MM, and the main cause of morbidity [2]. Therefore, bisphosphonates are used to prevent skeletal-related events. In addition, vitamin D plays an essential role in bone mineralization by enhancing intestinal calcium and phosphate absorption, and a deficiency can negatively influence bone mineralization [3]. In addition, vitamin D is now more recognized in oncology, as it has shown to have anti-inflammatory, pro-apoptotic, and anti-angiogenic properties by activation of the vitamin D receptor [4, 5]. However, measurement and supplementation of vitamin D are not standard of care in MM patients, although known risk factors for vitamin D deficiency are common in the MM population, including higher age and insufficient sunlight exposure. Studies have demonstrated that inadequate vitamin D levels are frequent in MM patients [6–8].

Over the past decade, several novel agents have significantly improved overall survival in MM patients [9]. However, many of these agents are neurotoxic, especially thalidomide and bortezomib used in upfront and relapsed treatment regimes, making peripheral neuropathy (PN) a common and severe adverse event of treatment. In addition, PN can also be induced by the disease itself by deposits of the malignant plasma cells or nerve damage as a result of radicular or medullar compression [10–14]. More insight has recently been gained concerning the capacity of vitamin D to interfere with the pathogenesis of PN, as an association between vitamin D and PN has been found [6, 7, 15]. A suggested mechanism of action of vitamin D in preventing PN is the upregulation of the nerve growth factor [16]. A depletion of this factor was found in cancer patients who developed PN during treatment with bortezomib, thalidomide, or vincristine [17]. Other neuroprotective mechanisms of vitamin D include the protection of nerve cells by anti-inflammatory effects, reduction of intracellular calcium, or reactive oxygen species [18–20].

Both the role of vitamin D in bone mineralization and the possible role in PN prevention support further investigation to the achievement of adequate vitamin D levels in MM patients. Several studies in for example nursing home residents have shown the difficulty of reaching adequate 25-hydroxyvitamin D (25[OH]D) levels [21–23]. The impact of vitamin D_3_ supplementation on serum 25(OH)D levels in MM patients has not yet been studied.

The aim of the current study was to determine the effectiveness of a new vitamin D_3_ dosing regimen in obtaining adequate 25(OH)D levels in vitamin D–deficient MM patients. Furthermore, the secondary objective was to evaluate the effect of vitamin D_3_ supplementation on the prevalence and severity of PN.

## Methods

### Study design and participants

This multicenter, prospective, single-arm study was conducted between October 2018 and November 2019 in the teaching hospitals Medical Centre Leeuwarden and Deventer Hospital, the Netherlands. Vitamin D insufficiency was defined as 25(OH)D levels < 75 nmol/L [6, 7, 24, 25]. Patients with MM, aged ≥ 18 years, and with 25(OH)D levels < 75 nmol/L (= 30 ng/mL) at the start of the study were eligible for participation. Patients were enrolled regardless of whether they had previously, were currently, or were planned to initiate neurotoxic treatment for MM. Patients who already had been prescribed vitamin D_3_ by a healthcare specialist, or had contraindications for the use of vitamin D_3_ according to the Summary of Product Characteristics of cholecalciferol tablets or oral solution [26, 27], were excluded. These contraindications were the following: hypersensitivity to the active substance(s) or to any of the excipients; hypercalcemia (> 2.60 mmol/L) and/or hypercalciuria (> 8 mmol/24 h for 80 percentile method); nephrolithiasis and/or nephrocalcinosis (only for Cholecalciferol Mylan® [Canonsburg, PA, USA]); serious renal impairment (eGFR < 30 mL/min/1.73m^2^, only for D-Cura®); hypervitaminosis D (> 220 nmol/L [> 88 ng/mL]); or pseudohypoparathyroidism (only for D-Cura®) [26, 27].

In concordance with the Medical Research Involving Human Subjects Act, approval of this study was obtained by a Medical Ethics Committee. All patients provided written informed consent prior to participation. The study is registered in the Netherlands Trial Register (NL6678).

### Prescription of vitamin D and adherence

After receiving informed consent, the patients’ hematologists prescribed vitamin D_3_, and the oral solution and tablets of vitamin D_3_ were distributed by the researchers. The general practitioner and community pharmacy were informed about the participation in the study, and the use of vitamin D_3_. Also, the community pharmacies were asked to verify that the patients were not taking vitamin D_3_ supplementation concomitantly. Furthermore, all patients confirmed in the consent form that no over-the-counter supplements containing vitamin D were used.

Therapy adherence was assessed at the visits after 2 and 6 months, by counting the remaining vitamin D tablets. Patients were considered therapy adherent when they used > 90% of the prescribed amount of vitamin D for the given time period.

### Dose escalation regimen and measurements of vitamin D

**Fig. 1:**
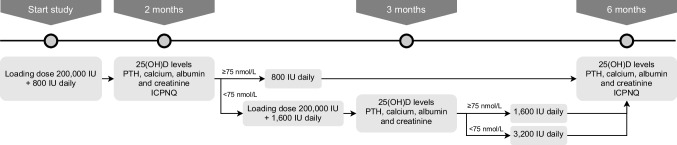
Study schedule with dose escalation regimen of vitamin D. 25(OH)D, 25-hydroxyvitamin D; ICPNQ, Indication for Common Toxicity Criteria Grading of Peripheral Neuropathy Questionnaire; PTH, parathyroid hormone

Figure 1 shows the study schedule with the dose escalation regimen for vitamin D_3_. The 3-level dose escalation regimen was designed based on the information that up to 10,000 IU orally every day for 5 months has been proven to be safe [24], and toxic effects were seen with doses of 40,000–100,000 IU per day for 1–2 months [27]. Before the start of the study, 25(OH)D levels were determined to assess an inadequate 25(OH)D level (< 75 nmol/L [= 30 ng/mL])). Serum levels of 25(OH)D were measured at baseline (*t* = 0), 2 (*t* = 2), 3 when dose adjustments at *t* = 2 were indicated (*t* = 3), and 6 (*t* = 6) months. All samples were analyzed in the laboratory of the Medical Centre Leeuwarden. A vitamin D_3_ loading dose (200,000 IU) was given at the start of the study, and vitamin D_3_ 800 IU tablets were regarded as the maintenance dose.

For safety reasons, calcium concentrations were determined after 1 month to verify that the calcium levels did not exceed 2.60 mmol/L. Based on the 25(OH)D serum level at *t* = 2 and *t* = 3 months, a second loading dose and/or maintenance doses of 800, 1600, or 3200 IU/day were prescribed. Parathyroid hormone (PTH), calcium, creatinine, and albumin levels were simultaneously analyzed with 25(OH)D at *t* = 0, *t* = 2, and *t* = 6 months, to ascertain the absence of contraindications for the use of vitamin D.

During each visit, patient-reported side effects related to vitamin D were inventoried. If side effects were present or one of the contraindications was met, vitamin D supplementation was terminated.

### Peripheral neuropathy assessment

To determine the PN grading, the “Indication for Common Toxicity Criteria Grading of Peripheral Neuropathy Questionnaire” (ICPNQ) was used [28]. This questionnaire is validated to distinguish different PN grades in MM patients, in which grade 0 means no PN and grade 3 means severe neuropathy where activities of daily living can no longer be performed independently. The ICPNQ was completed by one of the researchers in consultation with the patients at *t* = 0, *t* = 2, and *t* = 6 months. The researchers were blinded for the results of the 25(OH)D measurements. Furthermore, the cumulative dose of anti-myeloma therapy received during the study period was calculated. Any dose adjustment of anti-myeloma therapy was at the discretion of the treating hematologist.

### Statistical analysis

Descriptive statistics were used for the baseline characteristics. Data are presented as means (and standard deviation [SD]) or as medians (and interquartile range [IQR]). We planned to enroll 40 patients. We assumed an effect, i.e., an adequate 25(OH)D level, of the intervention in 60% of our patients. Given this assumption, the exact (Clopper-Pearson) 95% confidence interval would lie between 43 and 75%. With this, an effect of less than 43% could be excluded.

To determine the primary outcome, the proportion of patients in the Per Protocol population with an adequate 25(OH)D level (75–220 nmol/L [= 30–88 ng/mL]) after 6 months was determined. In addition, the one-sample *t* test was applied to evaluate the change in 25(OH)D levels after 6 months of vitamin D_3_ supplementation, and for the change in calcium, creatinine, and PTH levels after 2 and 6 months. For skewed distributions, i.e., delta 25(OH)D levels after 2 months and delta albumin after 2 and 6 months, the one-sample Wilcoxon signed-rank test was used. The McNemar-Bowker test was utilized to compare the symmetry of PN grades at *t* = 0 and *t* = 6 months. A *P* value < 0.05 indicated statistical significance. All analyses were performed using IBM SPSS Statistics 24.

## Results

**Fig. 2:**
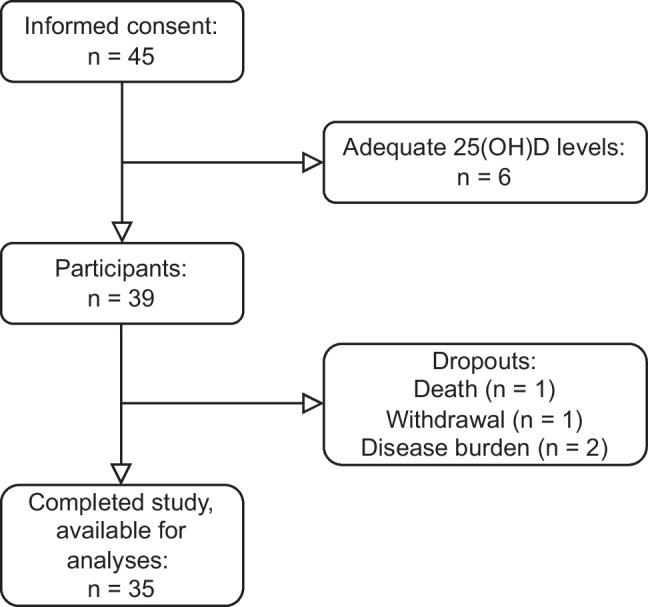
Study flow chart

Forty-five patients signed informed consent for this study. Figure 2 displays the flow chart of the study participants. Thirty-nine MM patients with inadequate (< 75 nmol/L) 25-hydroxyvitamin D (25(OH)D) levels were included. Table 1 shows the baseline characteristics of these patients. The mean (SD) age at baseline was 68 (8) years, and 72% of the participants were male. At baseline, the median 25(OH)D level of our cohort was 38 (IQR 32–52) nmol/L. The 25(OH)D levels were within the ranges of < 25, 25–50, and 50–75 nmol/L for 10.3%, 64.1%, and 25.6% of the participants, respectively.

**Table 1:** Baseline characteristics of the study population

Characteristic	*n* = 39
Age, mean (± SD)	68 (± 8)
Gender, male; *n* (%)	28 (72)
Race, Caucasian; *n* (%)	37 (95)
Symptomatic MM; *n* (%)	36 (92)
Time since diagnosis; median (IQR) in months	46 (23–71)
Current or previous anti-myeloma therapy with PN-inducing drugs; *n* (%)	35 (90)
Diabetes; *n* (%)	8 (21)
Alcohol; *n* (%)	
None	19 (49)
1–3 per week	8 (21)
4–9 per week	7 (18)
≥ 10 per week	5 (13)

*IQR*, interquartile range; *MM*, multiple myeloma; *PN*, peripheral neuropathy; *SD*, standard deviation

### Vitamin D intervention

The treatment period of 6 months was completed by 35 patients. In none of the patients, the contraindications for the use of vitamin D_3_ were reached. In 16 patients, dosing regimen level 1 (one loading dose + 800 IU daily) was applied, 15 patients required dosing regimen level 2 (two loading doses + 1600 IU since *t* = 2 months), and in 4 patients dosing regimen level 3 (two loading doses + 3200 IU since *t* = 3 months) was followed.

Four patients did not complete the 6-month study period. One patient died of MM, and three patients withdrew from the study: two as a result of their disease burden; and one patient experienced severe muscle pain possibly related to the use of vitamin D. This patient used a maintenance dose of 800 IU vitamin D, and performed a de- and rechallenge on her own initiative, which improved and worsened the complaints, respectively. The other participants had minor (e.g., diarrhea, cramp) to no adverse events.

The median percentage of therapy adherence was 99.4% (IQR 92.6–100.0%). Of the 35 patients who completed the study period, 27 patients were > 90% therapy adherent. In addition, six patients were at least 80% adherent. The remaining two patients were adherent at *t* = 2 months, but forgot to bring their vitamin D tablets at *t* = 6 months. They reported to be therapy adherent during the treatment period and as a result were considered therapy adherent.

### Effectiveness of vitamin D dosing regimen

**Fig. 3:**
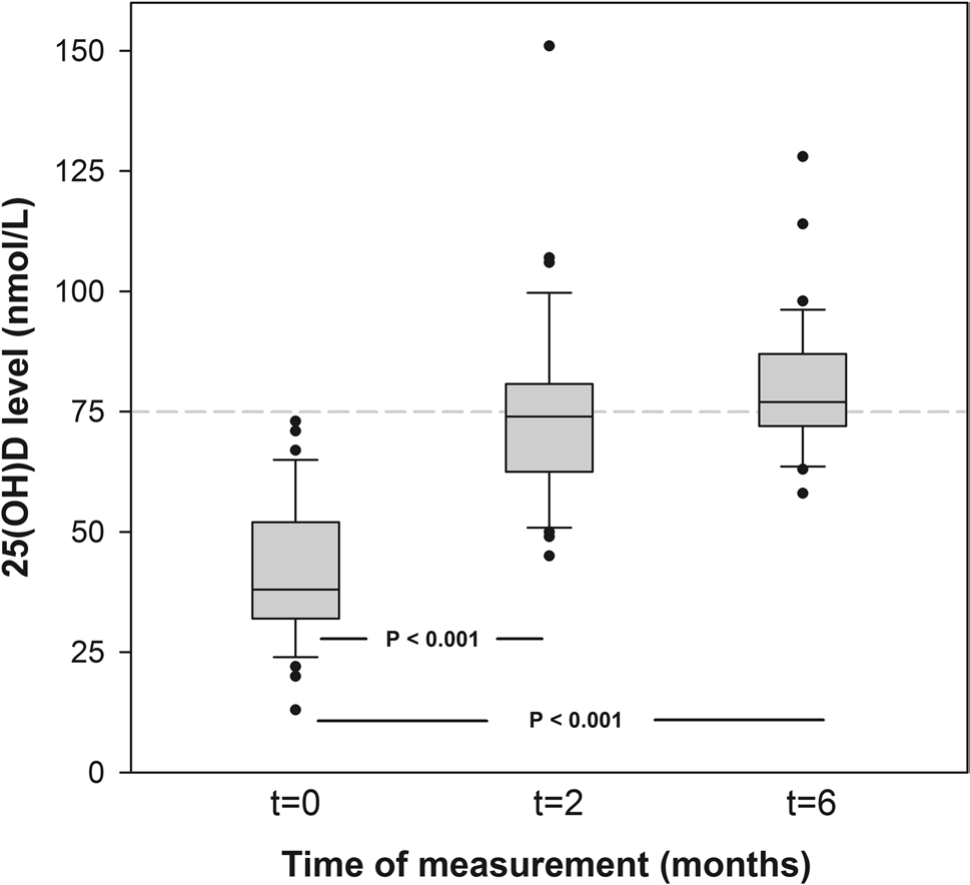
Boxplot displaying the distribution of 25(OH)D levels at baseline (*t* = 0), 2 (*t* = 2), and 6 (*t* = 6) months during treatment with vitamin D in 35 patients

The effect of the vitamin D_3_ dosing regimen on serum 25(OH)D levels is shown in Fig. 3. The median 25(OH)D level increased from 38 (IQR 32–52) nmol/L at *t* = 0 months to 77 (IQR 72–87) nmol/L at *t* = 6 months (*P* < 0.001). After 6 months, 66% of the subjects achieved a 25(OH)D level ≥ 75 nmol/L, and the remaining 34% had a 25(OH)D level of at least 58 nmol/L. None of the patients exceeded the maximum 25(OH)D reference value of 220 mmol/L. Half of the patients who did not achieve adequate 25(OH)D levels at the end of the study period received a maintenance dose of 800 IU vitamin D_3_ during the study, because of adequate 25(OH)D levels at 2 months.

As shown in Supplementary Table 1, no significant differences in mean or median calcium, albumin, and creatinine levels were observed in patients between *t* = 0 and *t* = 6 months during treatment with vitamin D_3_, and none of the patients exceeded normal reference values. PTH levels improved towards normal reference values (2–7 pmol/L) after 6 months.

### Peripheral neuropathy

Table 2 shows the distribution of PN grades 0 to 3 at *t* = 0 compared to *t* = 6 months in 35 patients who completed the study. The percentage of patients with any grade of PN decreased from 88.6% at baseline to 80% after 6 months. In 37% of the patients, the PN grade decreased, i.e., improved, after 6 months; in 60% of the patients, no change was observed; and in 3%, a worsening had occurred (*P* = 0.007). This effect was not yet seen after 2 months of treatment with vitamin D_3_ (*P* = 0.43).

**Table 2:**
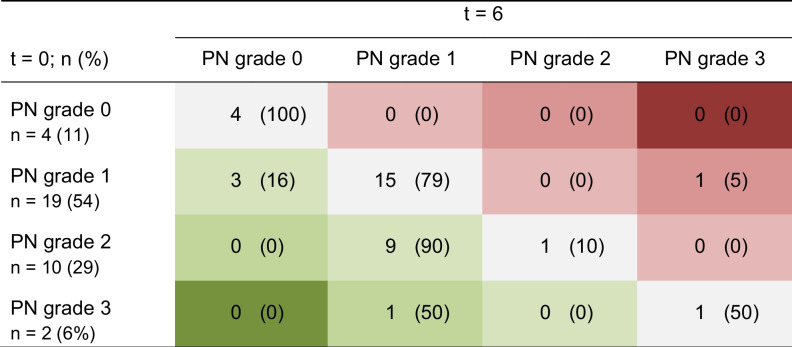
PN grades at baseline (*t* = 0) and after 6 months (*t* = 6) in 35 patients (*P* = 0.007). Green indicates an improvement of PN grade, and red indicates a worsening of PN grade

To analyze the influence of anti-myeloma therapy on PN grade, we divided the cohort into patients who had previously received neurotoxic treatment (*n* = 15) and patients who received neurotoxic treatment during the study (*n* = 17), as shown in Supplementary Table 2. Patients who did not receive any anti-myeloma treatment were excluded (*n* = 3). In the 17 patients who received treatment during the study, 1 patient had a worsening of PN (i.e., an increase in PN grade) and 6 patients experienced an improvement (i.e., a decrease in PN grade). In the 15 previously treated patients, 7 patients had an improvement in PN grade.

## Discussion

The designed dose escalation regimen significantly increased 25(OH)D levels in our study population. After 6 months of supplementation with vitamin D_3_, two-thirds of the patients achieved adequate 25(OH)D levels. In addition, the severity of PN for the whole group significantly decreased after 6 months.

The dose escalation regimen reached the predefined assumed effect of the intervention, i.e., adequate 25(OH)D levels in at least 60% of the patients. The regimen contained substantially higher vitamin D_3_ dosages than the advised daily dose of 800 IU vitamin D_3_ for risk groups in the Netherlands [29]. Nevertheless, the regimen appeared to be safe, without exceeding the upper end of the reference value for 25(OH)D, and with normal or improved calcium, creatinine, albumin, and PTH levels. Moreover, only minor adverse events were reported, and therapy adherence was high, which indicates that the regimen is feasible in patients diagnosed with and treated for MM.

The current study is the first to evaluate the impact of a vitamin D_3_ dosing regimen on serum 25(OH)D levels in MM patients. In a variety of other patient populations, different high-dose vitamin D_3_ regimens have been studied. In patients with breast, colorectal, lung, prostate, or pancreatic cancer, supplementation with 8000 IU/day of vitamin D_3_ resulted in an increase of 25(OH)D levels from 47.5 nmol/L at baseline to 90.5 nmol/L after 8 weeks. The target of 80 nmol/L was reached by 55.2% of the patients [21]. In nursing home residents, a loading dose of 200,000 IU and a maintenance of 100,000 IU every 13 weeks resulted in adequate 25(OH)D levels (≥ 75 nmol/L) in 58% of the individuals [22], and 8 weeks of supplementation with 1200 IU/day vitamin D_3_ in subjects with elevated waist circumference resulted in a decline of the percentage of subjects with 25(OH)D levels < 75 nmol/L of 64.5 to 61.3% [23]. Despite the differences in vitamin D_3_ dosing, these studies show the difficulty of reaching adequate 25(OH)D serum levels in all participants. Due to the small study population of our study, it was not possible to identify risk factors in MM patients for inadequate 25(OH)D levels after 6 months. Nevertheless, the low observed toxicity of vitamin D_3_ in this study, even in patients with 3200 IU of vitamin D_3_ as a maintenance dose, justifies supplementation with higher maintenance doses to achieve adequate 25(OH)D levels in all MM patients. It is recommended to monitor 25(OH)D levels in order to verify whether the applied dosing regimen results in target 25(OH)D levels.

The secondary objective of this study was to evaluate changes in the prevalence and severity of PN. The vast majority of our cohort, approximately 90%, experienced PN at baseline. Although the presence of PN did not change during vitamin D_3_ supplementation, a significant decrease in the severity of PN was observed at the end of the study period. These results should, however, be interpreted with caution, as our study design is not suitable to confirm the causality between 25(OH)D levels and PN. It is uncertain whether this improvement of PN was the result of higher 25(OH)D levels, or the result of time since nerve damage can be reversible. In addition, the use of PN-inducing agents and any dose adaptations were at the discretion of the treating physician and not controlled in our study. Patients who had previously received neurotoxic treatment were expected to have an improvement in neuropathy just due to passing of time, as opposed to patients who received neurotoxic treatment during the study, who would be expected to have a worsening of neuropathy. Nevertheless, only one of the 17 patients that received anti-myeloma treatment during the study had a worsening of PN grading, while 6 of the 17 patients had an improvement in PN grade. Although it is quite possible that vitamin D will no longer have an effect if the PN is too advanced, it could be that vitamin D has a protective effect at an earlier stage, thereby reducing the neuropathy. These observations indirectly support our hypothesis that adequate 25(OH)D levels could contribute to the prevention or improvement of PN [15].

Strengths of our study are the implementation of a strict dosing regimen, inclusion of patients in two hospitals situated in different areas of the Netherlands, and the uniform analysis of all blood samples in one laboratory. Furthermore, a validated questionnaire designed to measure PN grades in MM patients was used, and the questionnaire was always filled in by a trained researcher. This study was limited by the small study population, which makes it difficult to perform in-depth analyses.

This study implicates that substantially higher vitamin D doses than recommended by current guidelines are necessary to achieve adequate vitamin D levels in patients with multiple myeloma. Furthermore, our results suggest a possible role for vitamin D in PN treatment. Options to reduce the burden of PN are strongly needed in clinical practice. These observations warrant the execution of a randomized controlled trial to investigate the effect of vitamin D_3_ supplementation on the prevalence and severity of PN in newly diagnosed MM patients.

In conclusion, this dose escalation regimen proved to be a safe intervention to significantly increase serum 25(OH)D levels in MM patients. Two-thirds of the patients reached the target 25(OH)D serum level of ≥ 75 nmol/L. Furthermore, evaluation of PN at the end of the study period showed a significant decrease in the PN grading. However, this exploratory evaluation needs further confirmatory research.

## Supplementary Information

Below is the link to the electronic supplementary material.

Supplementary file1 (DOCX 20 KB)
