# Supplementary material for: Effectiveness of a vitamin D regimen in deficient multiple myeloma patients and its effect on peripheral neuropathy
Source: Support Care Cancer. 2023 Jan 26;31(2):138. doi: 10.1007/s00520-023-07574-0 (PMC9879810; doi:10.1007/s00520-023-07574-0)
Supplement: Supplementary file 1 — Supplementary file1 (DOCX 20 KB) [file 520_2023_7574_MOESM1_ESM.docx]

# Effectiveness of a vitamin D regimen in deficient multiple myeloma patients and its effect on peripheral neuropathy

Berdien E. Oortgiesen PharmD, PhD1; Marloes Dekens PharmD1; Ruud Stapel BSc1; Abdulrazzaq Alheraky PharmD1; Pauline Dannenberg - de Keizer BSc2; Claire Siemes MD, PhD2; Frank G.A. Jansman PharmD, Prof. dr3,4; Robby E. Kibbelaar MD, PhD5; Nic J.G.M. Veeger PhD6,7; Mels Hoogendoorn MD, PhD8,#; and Eric N. van Roon, PharmD, Prof. dr.1,3,#

Supplementary Table 1. Biochemical parameters at baseline (t = 0), after two (t = 2) and six months (t = 6) of intervention with the vitamin D dose escalation regimen in 35 patients.

| Biochemical parameters  (reference values) ^28^ | t = 0 | | t = 2 | | t = 6 | | P value  t0 → t2 | P value  t0 → t6 |
| --- | --- | --- | --- | --- | --- | --- | --- | --- |
| Calcium (2.10-2.55 mmol/L)^a^ | 2.3 | ± 0.1 | 2.3 | ± 0.1 | 2.3 | ± 0.1 | 0.75 | 0.13 |
| Albumin (35-55 g/L)^b^ | 34 | (32-37) | 34.5 | (32-37) | 36 | (33-38) | 0.281 | 0.192 |
| Creatinine (45-100 µmol/L)^a^ | 92 | ± 31 | 95 | ± 32 | 93 | ± 30 | 0.21 | 0.99 |
| PTH (2-7 ρmol/L)^a^ | 7.7 | ± 3.2 | 6.1 | ± 3.2 | 6.4 | ± 3.5 | 0.006 | 0.003 |

^a^Mean ± standard deviation

^b^Median (interquartile range)

Supplementary Table 2. Difference in PN grade after six months of intervention with the vitamin D dose escalation regimen in 32 patients, divided into patients who had previously received neurotoxic treatment and patients who received neurotoxic treatment during the study.

|  | Improvement | | Difference in PN grade  at t = 6 months | | | Worsening | |
| --- | --- | --- | --- | --- | --- | --- | --- |
|  | PN -2 | PN -1 | | PN 0 | PN +1 | | PN +2 |
| Previously treated | 1 | 6 | | 8 | 0 | | 0 |
| Treated during study | 0 | 6 | | 10 | 0 | | 1 |
